# A Practical Approach to the Differential Diagnosis of Intracranial Tumors: Gross, Histology, and Immunoprofile-based Algorithm

**DOI:** 10.7759/cureus.6384

**Published:** 2019-12-15

**Authors:** George S Stoyanov, Lilyana Petkova, Deyan L Dzhenkov

**Affiliations:** 1 General and Clinical Pathology, Forensic Medicine and Deontology, Medical University of Varna, Varna, BGR

**Keywords:** glioblastoma, tumors of the central nervous system, scherer figures, diagnostic algorithm, immunohistochemistry, pathology

## Abstract

Intracranial tumors are a diverse group of conditions, both benign and malignant, primary and metastatic and always require detailed medical information, radiological reports and deep knowledge of the histological hallmarks and immunohistochemical profile of different types of tumors and tumor-like processes. Despite it clinically often being possible to differentiate between primary and metastatic tumors, based on the tumor location and age of the patient, histological variants and rare tumor entries should always be considered in the histological differential diagnosis. A thorough diagnostic algorithm based on the location of the tumor and its histological features, together with some common pitfalls in immunohistochemical profiling, based on the 2016 revised World Health Organization (WHO) classification of tumors of the central nervous system should be implemented in all cases. Such an algorithm is especially valuable in cases where only small tumor fragments are sent for morphological evaluation, such as in deep parenchyma tumors. In these instances where only small fragments of the tumor are present for histology, some key features, corresponding to the WHO grade, may easily be missed or underreported. Furthermore, the histological verification of the tumor entry is the first, often overlooked, step in defining the presence or absence of WHO grade-specific mutations.

## Introduction and background

The differential diagnosis (DD) of intracranial tumors (ICTs) is a dubious task, requiring a highly trained neuropathologist and a set of both classical and immunohistochemical (IHC) markers for pinpointing the correct diagnosis [1]. The histological verification of the tumor entries is key in determining the optimal genetic tests needed for subtyping as per the revised 2016 World Health Organization criteria (WHO) and providing the patients with the optimal treatment [1].

## Review

**Figure 1:**
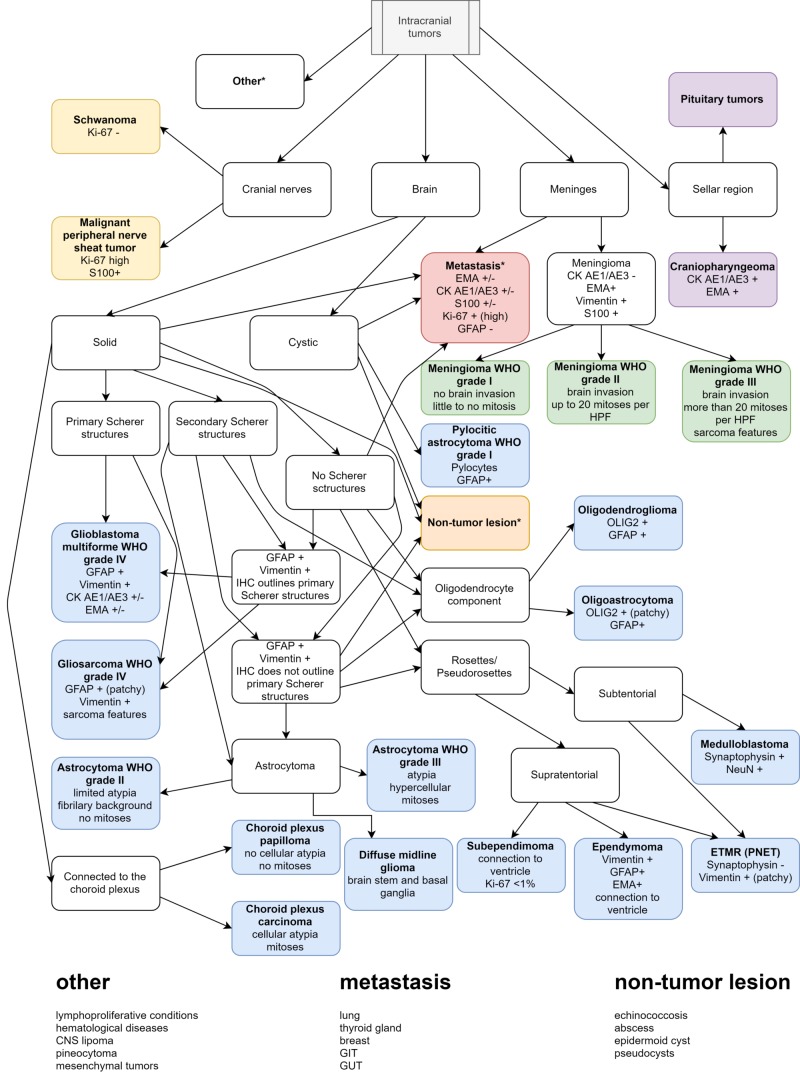
Diagnostic algorithm based on gross and histological features, with key IHC markers IHC: immunohistochemistry; WHO: World Health Organization; GFAP: glial fibrillary acidic protein; CK AE1/AE3: pan-cytokeratin; EMA: epithelial membrane antigen; HPF: high-power field; OLIG2: oligodendrocyte transcription factor; ETMR: embryonal tumor with multilayered rosettes; PNET: primitive neuroectodermal tumor; GIT: gastrointestinal tract; GUT: genitourinary tract; CNS: central nervous system

Before the morphological evaluation, the surgical report of gross tumor characteristics, place of origin of the tumor, together with the aid of any radiological report available, can be very suggestive of different tumor entries (Figure 1) [2].

Despite morphology on hematoxylin and eosin (H&E) being highly suggestive in some cases, often IHC is also needed to distinguish between different entries, especially in cases of only small tumor fragments being sent for pathological investigation. Although IHC is a useful tool in the DD of any lesion, when regarding ICTs, it is especially important to avoid some common pitfalls due to antigen mimicry such as the glial fibrillary acidic protein (GFAP) mimicry with epithelial membrane antigen (EMA) and the pan-cytokeratin (CK AE1/AE3), where if all three reactions are positive, only the GFAP one should be considered valid [3-6].

Tumors of the cranial nerves

**Figure 2:**
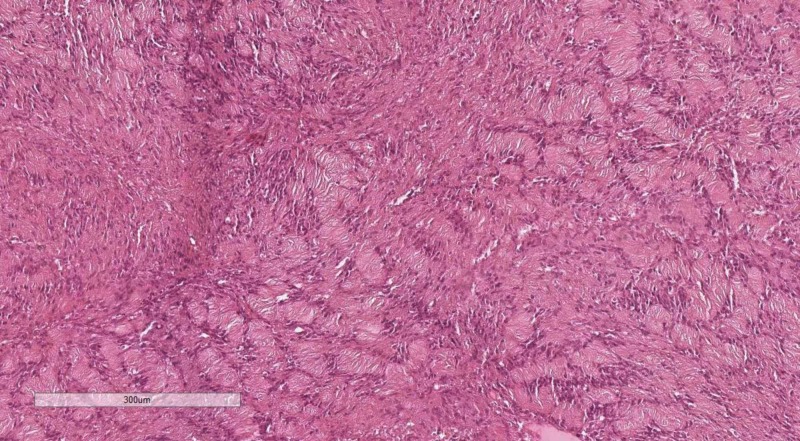
Typical appearance of Verocay bodies in Schwannoma. H&E stain, original magnification 100x H&E: hematoxilyn and eosin

Cranial nerve tumors are relatively rare, among other ICTs. The two main entries in this category are Schwannoma - a benign tumor and malignant peripheral nerve sheath tumor (MPNST) - its malignant counterpart [7]. Schwannomas classically present with Antony A and B zones, with pathognomonic Verocay bodies (Figure 2). However, when histologically verifying on small tissue fragment, sometimes the DD between the two entries is difficult. In this case, an IHC stain with Ki-67 is useful - Schwannomas do not give a positive reaction, whilst MPNSTs have a high percentage of positive nuclei. MPNSTs are also p53 positive on IHC.

Tumors of the sellar region

**Figure 3:**
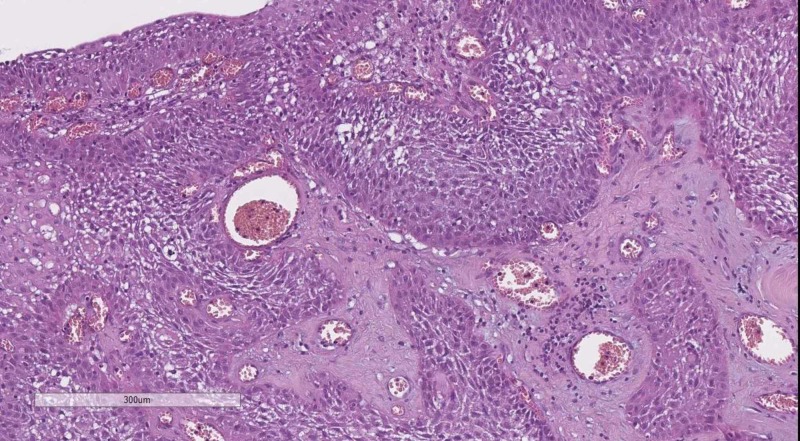
Typical appearance of craniopharyngioma. H&E stain, 100x H&E: hematoxylin and eosin

Often an underlooked region, when considering ICTs as again surgical and radiological reports may prove highly informative, especially in cases where the patient has abnormal hormone levels, further pointing to the type of adenoma of the pituitary [8]. Even in such cases, it is useful to also consider pituitary carcinomas, which have a high Ki-67 percentage of positive nuclei, unlike adenomas and also craniopharyngiomas - a disgerminative squamous cell tumor positive for EMA, CK AE1/AE3 and p53 (Figure 3) [9].

Tumors of the meninges

Meningeal tumors can pose a difficulty in the DD, especially the more anaplastic types which may also have deep invasion into the brain. Also important to consider here is the surgical and radiological report. If the tumor is diffuse then it is much more likely to be a distant metastasis. Such tumor involvement from the meninges have the IHC profile of the primary lesion and generally EMA and CK AE1/AE3 positive with a high Ki-67 index.

**Figure 4:**
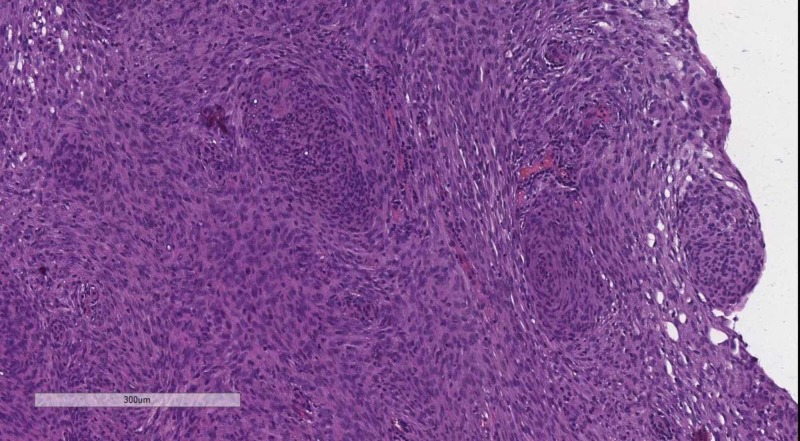
WHO grade I meningioma - no mitotic figures or brain parenchyma invasion. H&E stain original magnification 100x WHO: World Health Organization; H&E: hematoxylin and eosin

**Figure 5:**
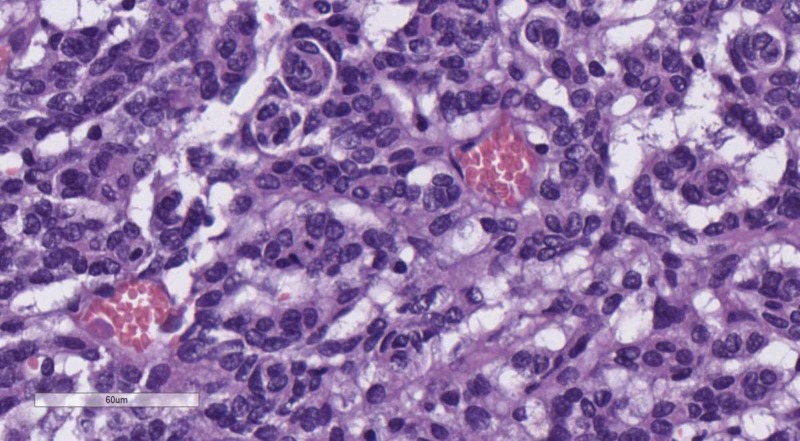
WHO grade II meningioma. H&E stain original magnification 400x WHO: World Health Organization; H&E: hematoxylin and eosin

**Figure 6:**
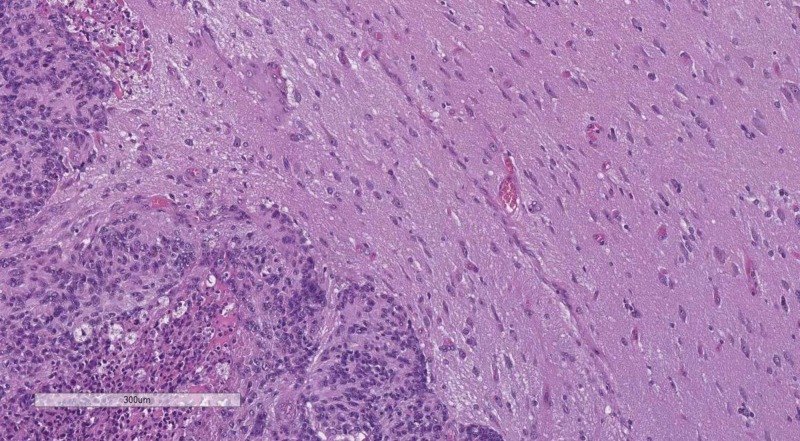
WHO grade III meningioma with brain parenchyma invasion. H&E stain original magnification 100x WHO: World Health Organization; H&E: hematoxylin and eosin

Classical tumors of the meninges are the meningioma group, graded WHO, based on their histological profile. WHO grade I tumor (Figure 4) present with minimal to no cellular atypia, no mitotic figures and do not invade the brain parenchyma, while WHO grade II (Figure 5) meningiomas show brain parenchyma invasion and may present with up to 20 mitotic figure per 10 high-power fields (HPF) [10-12]. WHO grade III meningiomas (Figure 6) also have brain parenchyma invasion, cellular atypia, more than 20 mitotic figures per 10 HPF or may even have a sarcomatoid differentiation [2].

Useful marker in the differentiation between metastasis and meningiomas, regardless of WHO grade are IHC marking with vimentin and S100, with up to 70% showing positive reaction with EMA, without a reaction for CK AE1/AE3 [11].

Tumors originating from the choroid plexus

**Figure 7:**
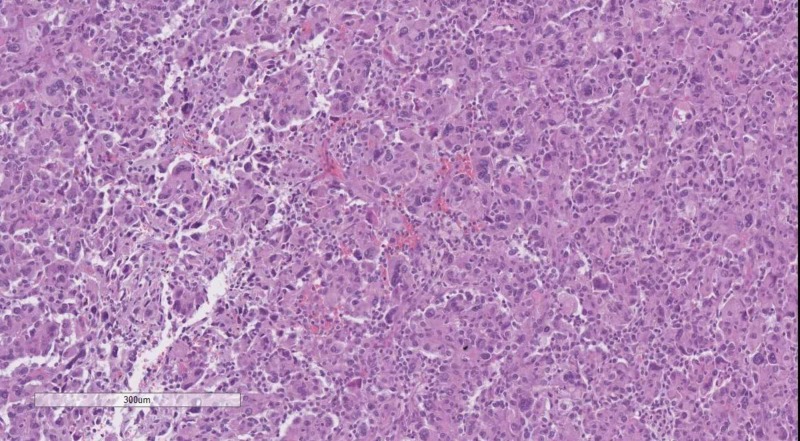
Choroid plexus carcinoma. H&E stain, original magnification 100x H&E: hematoxylin and eosin

Normally these tumors present in the pediatric population, with some of them being congenital. The two main entries here are the choroid plexus papilloma (CPP) - a benign entry and its malignant counterpart - choroid plexus carcinoma (CPC) [13]. DD between the two should be considered on histological criteria alone with CPP having no cellular atypia and mitotic figures, highly similar to a normal choroid plexus, with more hyperchromic cytoplasm due to glycogen accumulation, whilst CPC has cellular atypia to some extent and presence of mitotic figures (Figure 7) [14].

Tumors of the brain parenchyma

The biggest group, both based on incidence and individual entries. The DD here can be especially difficult, with the lack of medical information regarding the condition of the patient and in some cases the inability of the patient to share information regarding his past condition. Even in cases with a patient with a well known distant malignancy, the possibility of synchronous central nervous system (CNS) tumor should always be considered.

Whilst metastatic conditions often present as well-circumscribed masses, often multiple, they may present as solitary lesions mimicking the gross and radiological appearance of a primary tumor.

When considering the DD of such lesion it is best to start the diagnostic process with their gross appearance.

Cystic lesions

**Figure 8:**
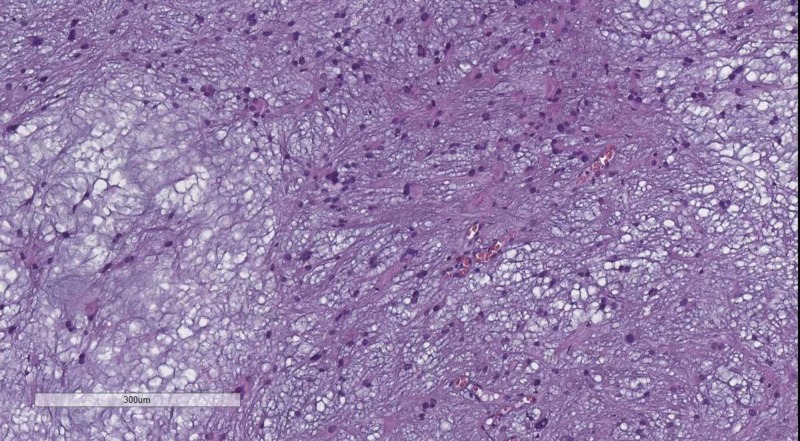
Pilocytic (WHO grade I) astrocytoma, H&E stain, original magnification 100x WHO: World Health Organization; H&E: hematoxylin and eosin

A relatively small percentage of brain tumors. The highest rate of cystic tumors is attributed to CNS metastasis, which presents with the IHC profile of the primary lesion and is generally EMA and CK AE1/AE3 positive, with a high Ki-67 index. Another entry in this group is the WHO grade pilocytic astrocytoma (Figure 8) - a tumor with astrocytic glial differentiation, presenting as a pilocytic rich, GFAP positive lesion, predominantly in the pediatric population. WHO grade I astrocytomas are an important factor for the further development of higher WHO grade glial tumors [2]. Furthermore, it is important to also note the rich amount of pathological processes in the CNS that can present as cystic lesions, both primary and secondary, such as pseudocysts, echinococcosis, abscesses or epidermoid cysts.

Solid lesions

**Figure 9:**
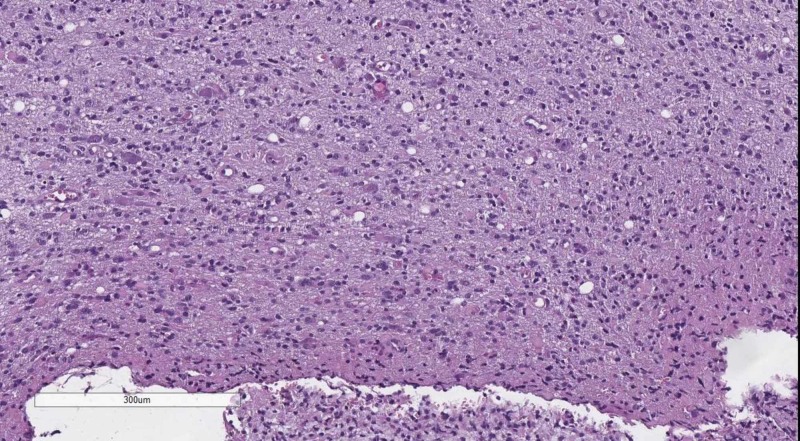
Diffuse (WHO grade II) astrocytoma, H&E stain, original magnification 100x WHO: World Health Organization; H&E: hematoxylin and eosin

**Figure 10:**
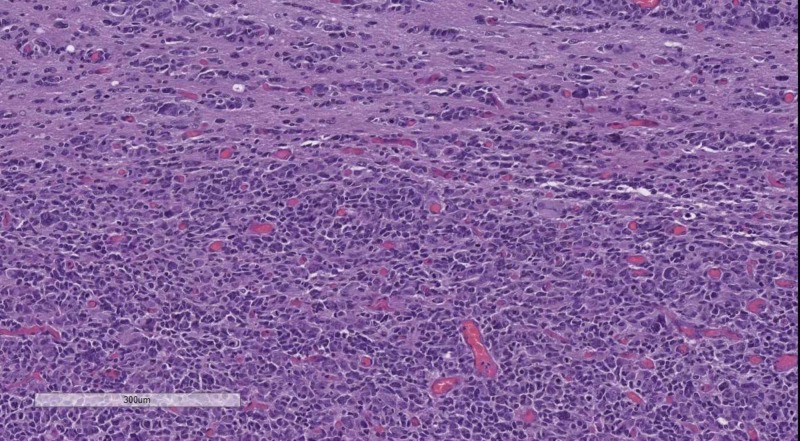
Anaplastic (WHO grade III) astrocytoma, H&E stain, original magnification 100x WHO: World Health Organization; H&E: hematoxylin and eosin

DD of solid brain parenchyma lesions should always start with the depiction or lack thereof of Scherer structures. Hans Joachim Scherer was a German pathologist, devout to the pathology of CNS tumors and established a wide set of histological criteria, based on a hematoxylin and eosin (H&E) stain, which are the basis of the WHO grading of glial tumors (Figures 9-10) [15-18].

**Figure 11:**
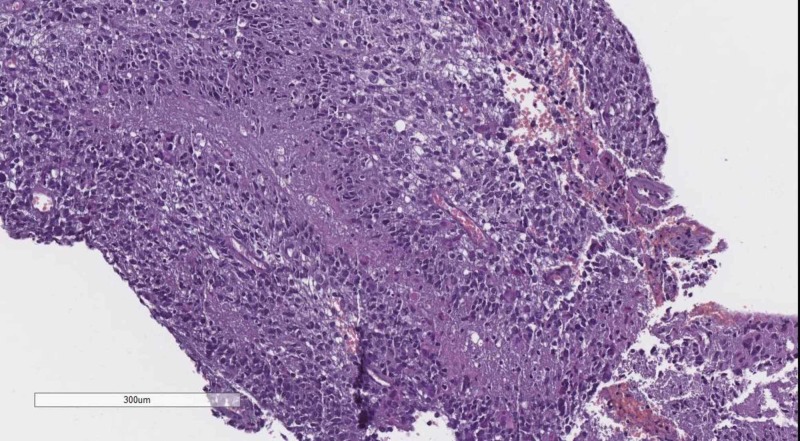
Primary Scherer figure (pseudopalisadic necrosis), H&E stain, original magnification 100x H&E: hematoxylin and eosin

**Figure 12:**
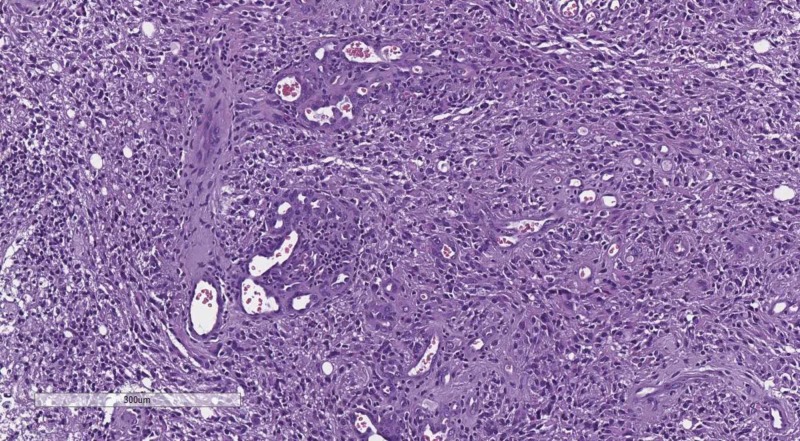
Glomeruloid vascular proliferation, H&E stain, original magnification 100x H&E: hematoxylin and eosin

**Figure 13:**
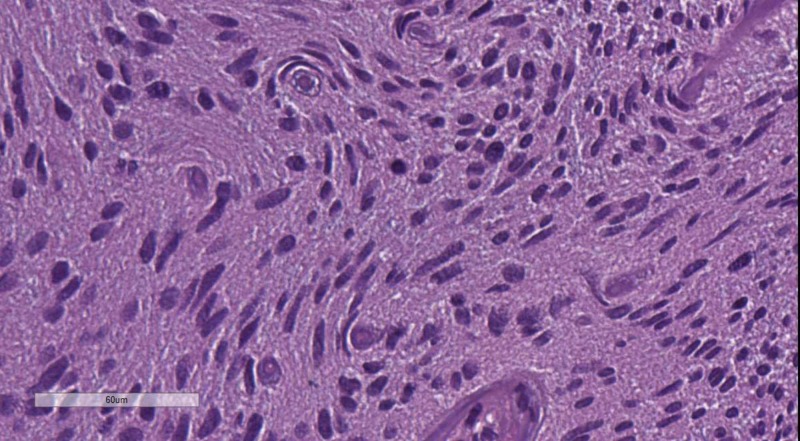
Perineural satelitosis, H&E stain, original magnification 400x H&E: hematoxylin and eosin

**Figure 14:**
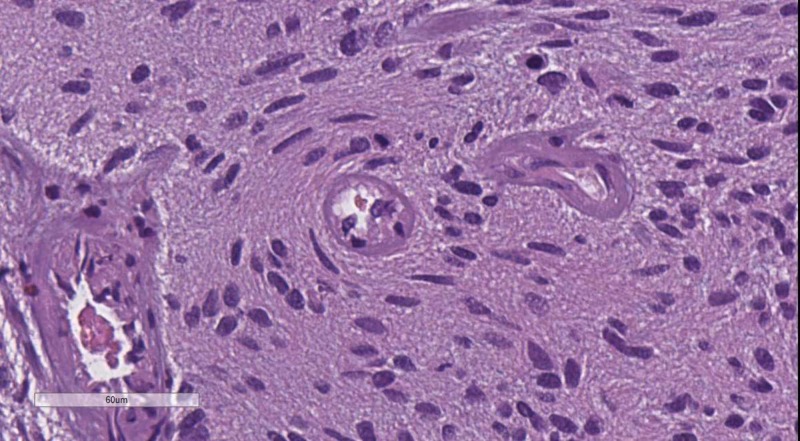
Vascular satelitosis, H&E stain, original magnification 400x H&E: hematoxylin and eosin

**Figure 15:**
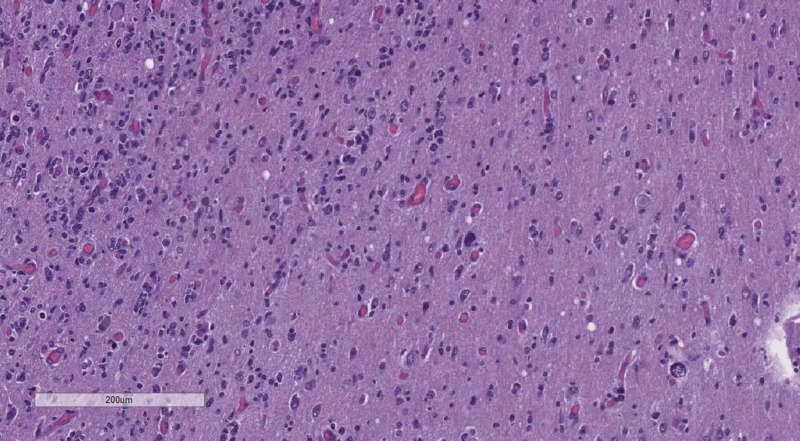
Tractal aggregation, H&E stain, original magnification 200x H&E: hematoxylin and eosin

**Figure 16:**
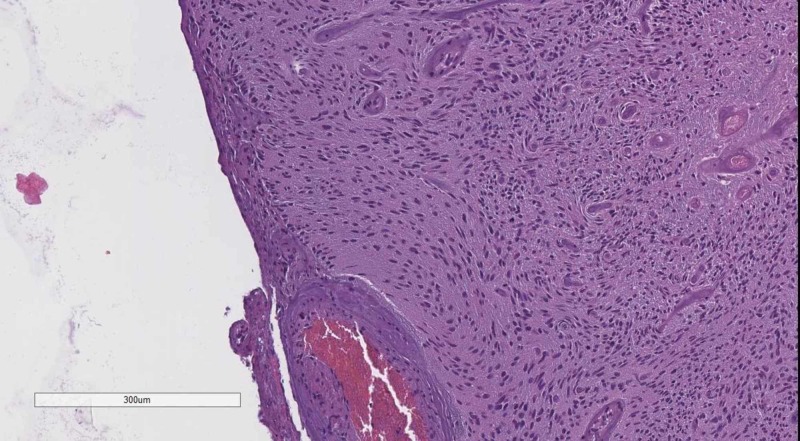
Subpial aggregation (palisading), H&E stain, original magnification 100x H&E: hematoxylin and eosin

Important to consider here are the so-called primary Scherer structures - foci of pseudopalisading necrosis, pathognomonic for glioblastoma multiforme (GBM), a WHO grade IV astrocytic glial neoplasm (Figure 11). Secondary Scherer structures, as described by Scherer, are histological patterns based on the spread, growth and biological potential of glial tumors. These include subpial palisading of tumor cells, satellitosis of tumors cell around preserved neurons and blood vessels, tractal aggregation of tumors cells and the nearly pathognomonic glomeruloid vascular proliferation (Figures 12-16). Key here are the patterns of growth of glial neoplasm, although aggressive in their growth and clinical course, these tumors take a long time to destroy the normal brain structures, unlike lower WHO grade meningiomas for example, and therefore as a definition present late clinically with an excessive in size tumor mass. The glomeruloid vascular proliferation, although being a factor of tumor growth and a distinct phenomenon from the other, should also be considered together with them as a key feature pointing towards a glial tumor, regardless of the WHO grade.

As already mentioned primary Scherer figures are pathognomonic for GBM, with secondary Scherer figures also being present, although rarely being reported on histology. GBMs show a distinct IHC profile with GFAP, Vimentin and S100 positivity. It is important to consider that around 2% of GBM lose their IHC reaction with GFAP, whilst preserving their vimentin reaction. Also key to note here is the already mentioned antigen mimicry reactions with EMA and CK AE1/AE3 [5]. Like all other glial tumors with astrocytic differentiation, GBM has a varying Ki-67 index, which cannot be used to determine the WHO grade alone.

**Figure 17:**
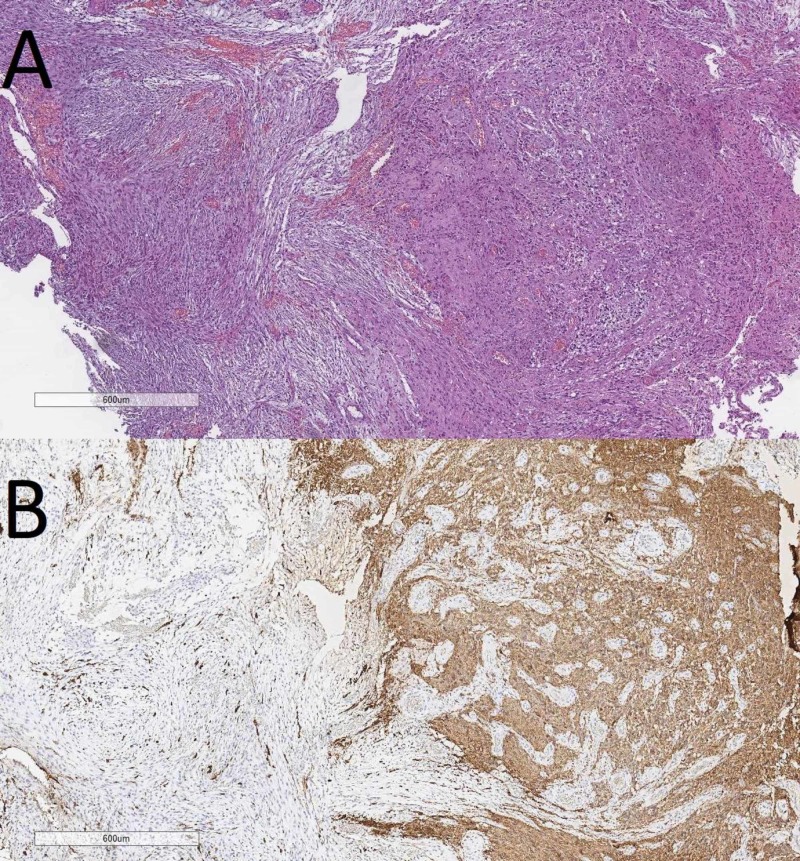
Gliosarcoma, (A) H&E stain, original magnification 40x and (B) GFAP IHC stain, original magnification 40x H&E: hematoxylin and eosin; GFAP: glial fibrilary acidic protein; IHC: immunohistochemistry

An often underdiagnosed tumor also presenting with primary and secondary Scherer structures is gliosarcoma, a WHO grade IV tumor. Gliosarcoma (Figures 17) although a distinct entry, shares most of its features with GBM. However, the presence of a mesenchymal component with sarcomatoid features is key. Also important here is the IHC profile diffusely positive for vimentin, with a patchy expression for GFAP, only in the glial component of the tumor.

If there are no primary Scherer structures on H&E, this does not exclude the diagnosis of GBM. Tumors rich in secondary Scherer structures should be subjected to IHC evaluation, as IHC with GFAP especially may outline the poorly defined pseudopalisading necrotic foci. If such is not outlined by IHC, then a diagnosis of a glial tumor WHO grade II or III should be considered. If located on the midline or the basal ganglia, the diagnosis of diffuse midline glioma should be considered, regardless of WHO criteria adherence to an individual grade.

Astrocytomas WHO grade II (Figure 9) are GFAP positive, show limited cellular atypia, a fibrillary background, and no mitotic figures, whist astrocytomas WHO grade III (Figure 10) have cellular atypia, although limited, are densely hypercellular and may present with abundant mitotic figures [2].

**Figure 18:**
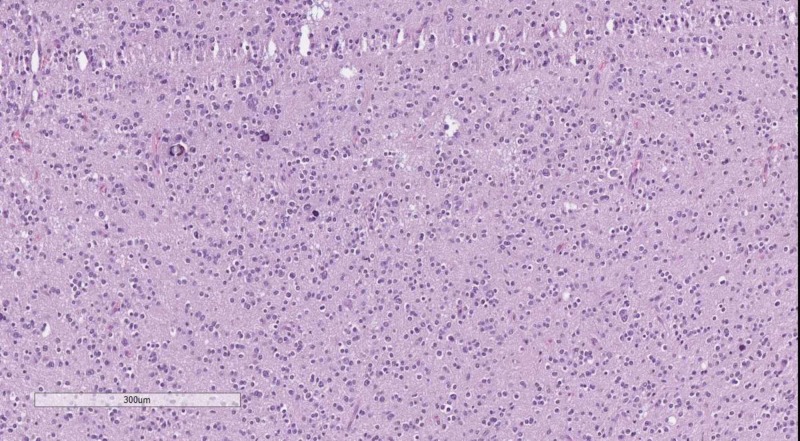
Oligodendroglioma, H&E stain, original magnification 100x H&E: hematoxylin and eosin

The absence of Scherer figures on H&E and IHC should sway the pathologist in the direction of more rare primary entries or metastasis if GFAP negative. The presence of a distinct oligodendrocyte-like component of the tumor should point to either oligodendroglioma (Figure 18) - GFAP and (oligodendrocyte transcription factor) OLIG2 positive or oligoastrocytoma - OLIG2 expression in the oligodendrocytic component and diffuse GFAP expression, stronger in the astrocytic component.

The absence or limited amount of Scherer structures, with the absence of an oligodendrocytic component, should sway the pathologist to search for rosettes or pseudorosettes and again consult the surgical and radiological report [2,19,20].

**Figure 19:**
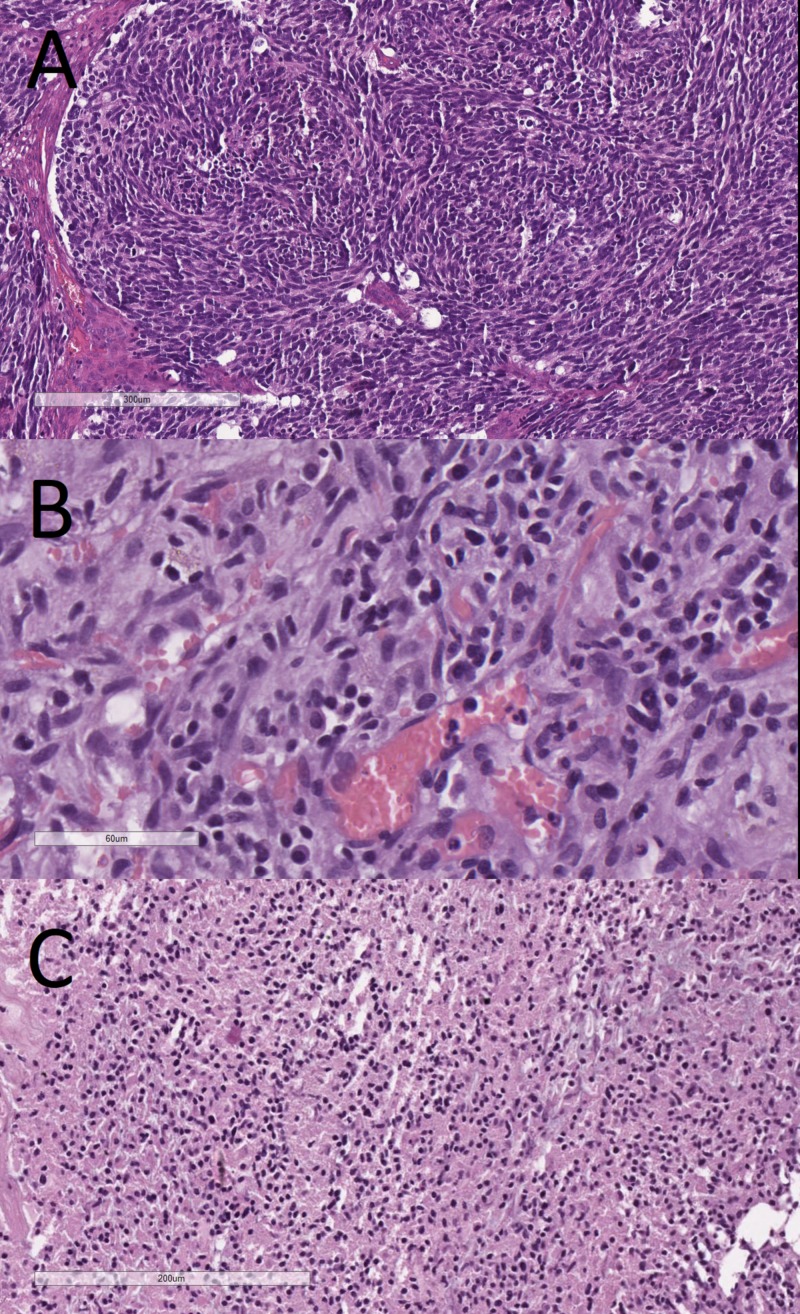
(A) Medulloblastoma, H&E stain, original magnification 100x; (B) ETMR, H&E stain, original magnification 400x; ependymoma, H&E stain, original magnification 200x; (C) Ependimoma, H&E stain, original magnification 200x H&E: hematoxylin and eosin; ETMR: embryonal tumor with multilayered rosettes

Subtentorial tumors with rosettes are predominantly medulloblastomas (Figure 19), which are synaptophysin and NeuN positive, with a minority of such subtentorial tumors being the embryonal tumors with multilayered rosettes (ETMR), previously referred to as primitive neuroectodermal tumors (PNET) (Figure 19) which are synaptophysin negative and have a patchy vimentin expression. ETMRs are much more common with a supratentorial location.

Other tumors with rosettes or pseudorosettes are the ependymoma (Figure 19) and subependymoma tumors, both of which have at least in some capacity a connection to the ventricular system. Generally on IHC ependymomas are vimentin, GFAP and EMA positive, while subependymomas despite their varying and non-specific IHC profile have Ki-67 index of <1%.

Metastasis

The most common metastatic entries to the CNS are from the lungs, breast, gastrointestinal tract, genitourinary tract and melanomas [21,22]. Despite sometimes phenotypically different and hard to identify these lesions always preserve the IHC profile of the primary lesion, contrasting from the IHC profile of primary CNS tumors (Table 1).

**Table 1:** IHC profile of the most common ICT by differentiation * dependant on the type of metastasis; GFAP: glial fibrillary acidic protein; OLIG2: oligodendrocyte transcription factor; CK AE1/AE3: pan-cytokeratin; EMA: epithelial membrane antigene; SMA: smooth muscle actin

antibody	astrocytic	oligodendrocytic	meningeal	embryonal	metastasis*
GFAP	+	+	-	+/-	-
OLIG2	-	+	-	-	-
NeuN	in preserved neurons only	-	-	+/-	-
Synaptophysin	+	-	-	+	+/-
CK AE1/AE3	+/-	-	-	+/-	+/-
EMA	+/-	-	+	-	+/-
Vimentin	+	-	+	+/-	+/-
SMA	-	-	+	-	+/-
S100	+	+	+	+/-	+/-
Ki-67	variable	low	variable	high	high

Other tumors

**Figure 20:**
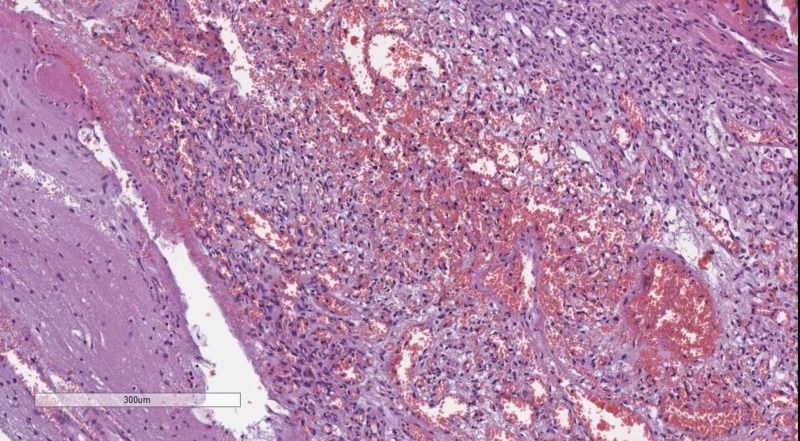
Hemangioblastoma, H&E stain, original magnification 100x H&E: hematoxylin and eosin

**Figure 21:**
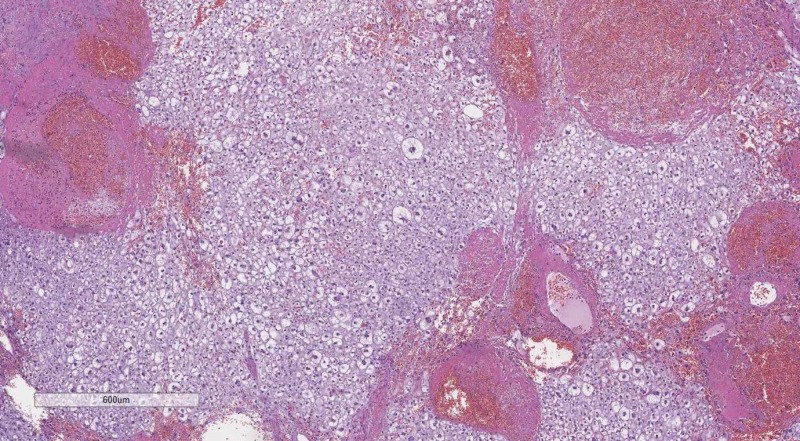
Cranial chordoma, H&E stain, original magnification 40x H&E: hematoxylin and eosin

**Figure 22:**
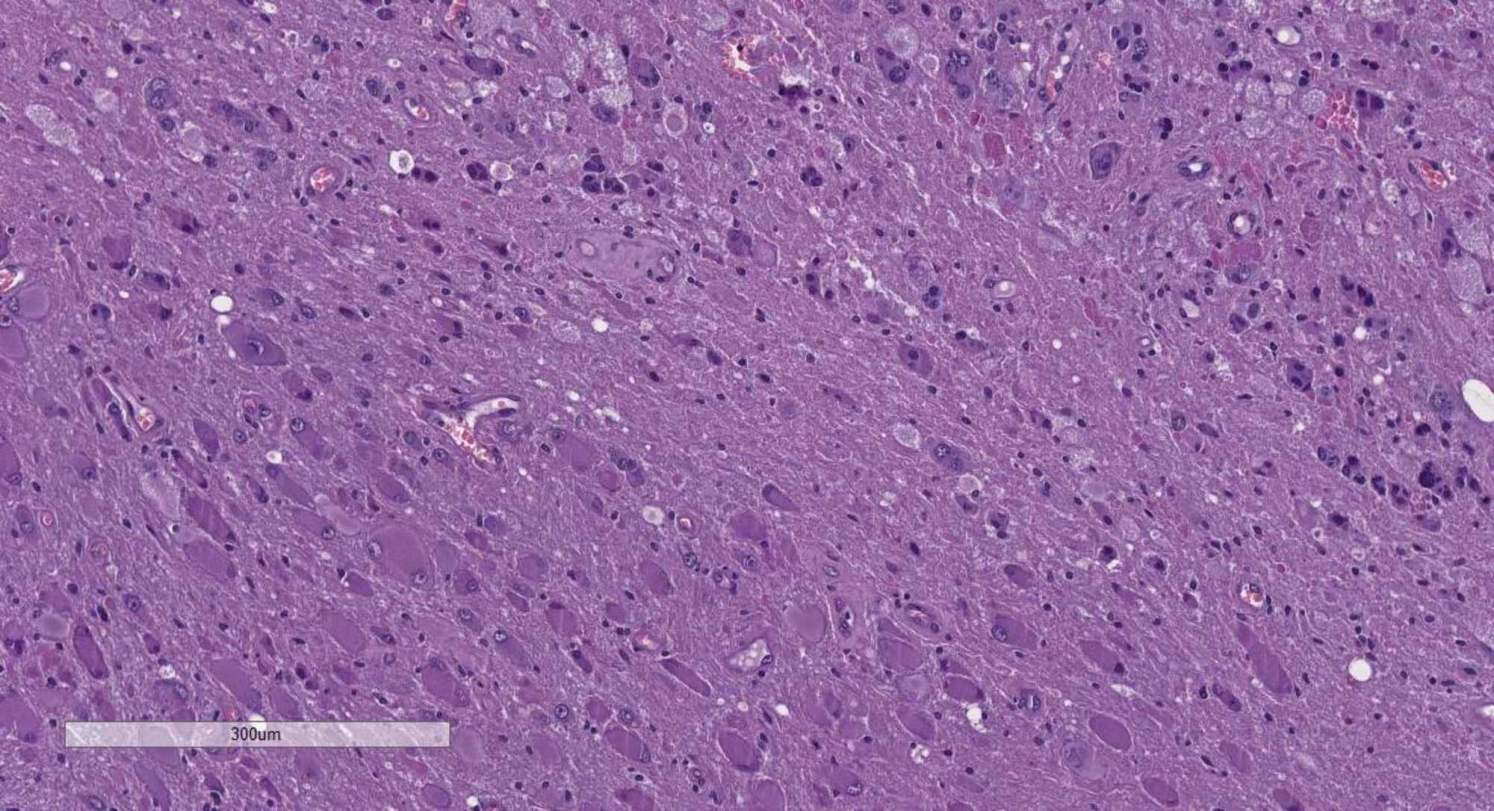
Ganglioglioma, H&E stain, original magnification 100x H&E: hematoxylin and eosin

**Figure 23:**
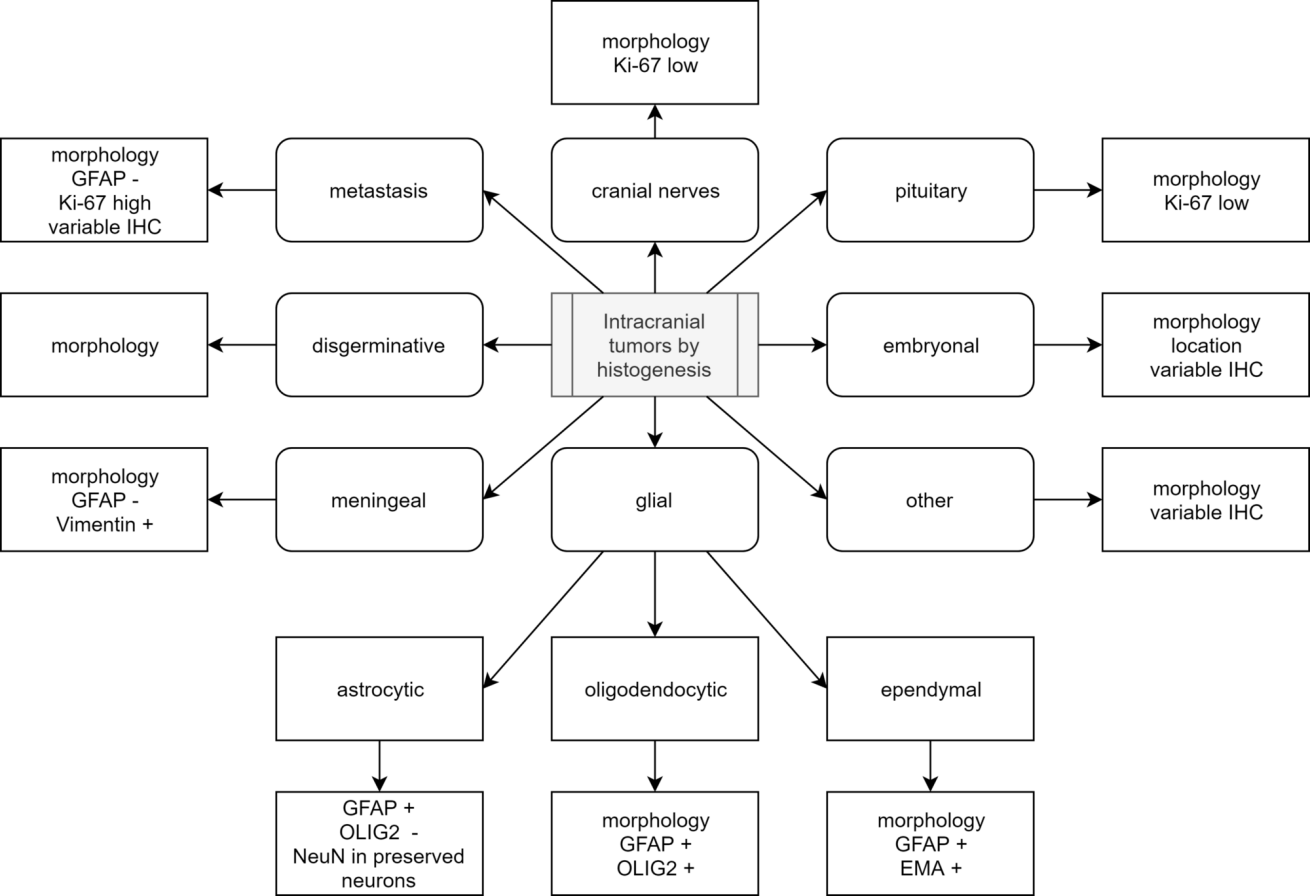
Diagnostic algorithm based on histogenesis of tumors OLIG2: oligodendrocyte transcription factor; GFAP: glial fibrillary acidic protein; EMA: epithelial membrane antigene; IHC: immunohistochemistry

Some rare tumors, which do not fit the above-mentioned criteria include infiltration or primary lymphoproliferative disorders, infiltration from hematological malignancies, mesenchymal tumors, pineocytoma and even easily diagnosable, although rare tumors such primary CNS lipoma (Figures 20-23) [23].

Primary tumor classification, according to WHO

As per the 2016 revision of the WHO classification of tumors of the CNS, even if histologically defined, the tumors should always be identified as not otherwise specified (NOS), unless IHC or genetic analysis is performed to identify the subgroup of mutations relevant to the specific tumor entry, such as isocitrate dehydrogenase (IHD), 1p/19q, and ATRX [2,20]. In rare instances, primary tumors may also be identified as not elsewhere classified (NEC) [24-28]. NEC should be used in cases when extensive genetic testing was performed on a specific histological entry, however, the result from the test does not allow for inclusion into any of the specified WHO diagnoses.

## Conclusions

The diagnosis and DD of intracranial tumors require a systematic approach, acquaintance with the clinical, surgical and radiological documentation of the patient and in-depth knowledge of the diagnostic criteria of the different groups of tumors. The diversity of the tumors, based both on their primary and metastatic origin, together with the diversity of the primary lesions, their criteria of WHO grading and varying IHC profile, together with the genetic alterations defined in the 2016 revision of the WHO classification makes the morphological diagnosis of CNS tumors a challenging task, requiring multiple modalities for defining the tumor type.
